# ZooPathWeb: a comprehensive web resource for zoonotic pathogens

**DOI:** 10.1093/bioadv/vbad094

**Published:** 2023-07-10

**Authors:** Rui-Si Hu, Xin Zhang, Yanming Wei

**Affiliations:** Yangtze Delta Region Institute (Quzhou), University of Electronic Science and Technology of China, Quzhou, Zhejiang 324003, China; Institute of Fundamental and Frontier Sciences, University of Electronic Science and Technology of China, Chengdu, Sichuan 610054, China; Yangtze Delta Region Institute (Quzhou), University of Electronic Science and Technology of China, Quzhou, Zhejiang 324003, China; Institute of Fundamental and Frontier Sciences, University of Electronic Science and Technology of China, Chengdu, Sichuan 610054, China; Yangtze Delta Region Institute (Quzhou), University of Electronic Science and Technology of China, Quzhou, Zhejiang 324003, China; School of Computer Science and Technology, Xidian University, Xi’an, Shaanxi 710071, China

## Abstract

**Motivation:**

Zoonotic pathogens, such as viruses, bacteria, fungi and parasites, can be transmitted from animals to humans, causing a wide range of diseases that can vary from mild to life-threatening. These pathogens typically exhibit a broad host range, infecting domestic and/or wild animals, which serve as reservoirs of infection. Human infection can occur through direct contact with infected animals or their body fluids, consumption of contaminated food or water, or via bites from infected arthropod vectors. Understanding the epidemiological characteristics and population structure of zoonotic pathogens is of paramount importance for preventing and controlling the spread of zoonotic diseases.

**Results:**

Here, we present ZooPathWeb, a comprehensive online resource for zoonotic pathogens. ZooPathWeb provides essential information on pathogens that are particularly relevant to public health and includes a literature collection organized by pathogen classification, such as lineage, host, country or region and publication year. Moreover, we have developed four web-based utility tools for this release: SeqNHandle, PaPhy-ML, TreeView and BLAST. These tools are specifically designed to facilitate the identification of population structure and adaptive evolution in relation to zoonotic pathogens.

**Availability and implementation:**

The ZooPathWeb website is accessed via http://lab.malab.cn/~hrs/zoopathweb/. The source code for AKINND, which is used for collecting pathogen-related literature, can be found at https://github.com/RuiSiHu/AKINND. Additionally, the source code for PaPhy-ML, utilized for phylogenetic analysis, can be found at https://github.com/RuiSiHu/PaPhy-ML.

**Supplementary information:**

Supplementary data are available at *Bioinformatics Advances* online.

## 1 Introduction

Human–animal contact poses an increased risk of zoonotic spillover events (Plowright *et al.*, 2017), wherein pathogens originating from vertebrate animals establish infections in humans, often leading to severe consequences due to the lack of prior immunity. The World Health Organization’s official report in 2020 highlights the identification of over 200 known types of zoonotic pathogens, significantly contributing to the transmission of epidemic diseases in humans. Notable examples include zoonotic viral diseases, such as SARS, monkeypox, avian influenza and rabies, bacterial diseases such as anthrax, tuberculosis, brucellosis, pestis and Q fever (Christou, 2011), as well as parasitic diseases, such as malaria, trypanosomiasis and toxoplasmosis (Esch and Petersen, 2013). Furthermore, global changes in land use resulting from urbanization, agricultural expansion and the destruction of natural habitats further escalate the risk of various zoonotic spillover events.

To investigate the molecular epidemiology of pathogens, it is crucial to understand the dynamics of diseases resulting from infections in reservoir hosts, including domestic and wild animals. Additionally, assessing the risk of pathogen exposure to human hosts is essential for enhancing surveillance efforts and implementing appropriate interventions. While extensive research has been conducted on the genetic variation of zoonotic pathogens and their association with reservoir hosts, there is a significant lack of systematic collection and classification of these datasets. Furthermore, there is an ongoing need to develop practical bioinformatics tools capable of swiftly identifying the molecular characteristics of pathogens, thereby facilitating molecular epidemiological studies.

In this study, we have established a comprehensive web resource called ZooPathWeb (Zoonotic Pathogen Webserver), which encompasses a wide range of zoonotic pathogens that are of significant public health importance. By utilizing the nucleic acid data available in the NCBI Nucleotide database, we have developed the AKINND (Acquire Key Information from NCBI Nucleotide Database) program using the C# programming language. With this program, we retrieved a total of 262 098 articles and created a literature search library using the Vue.js framework. Furthermore, in this version, we have introduced four web-based applications: SeqNHandle, PaPhy-ML, TreeView and BLAST.

## 2 Methods

ZooPathWeb was constructed using Django 2.0, a free and open-source framework, within the Python 3.7.1 environment. The web applications were tested on an Ubuntu platform and developed using MySQL 5.7.37 as the data storage system. All application tools and their corresponding backend programs were implemented in Python. The web interfaces and servers, based on HTML, underwent rigorous testing across multiple browsers, including Safari, Google Chrome and Firefox. More detailed information can be found in the Supplementary Material.

## 3 Results and discussion

### 3.1 Collection of empirical data

**Figure 1.**
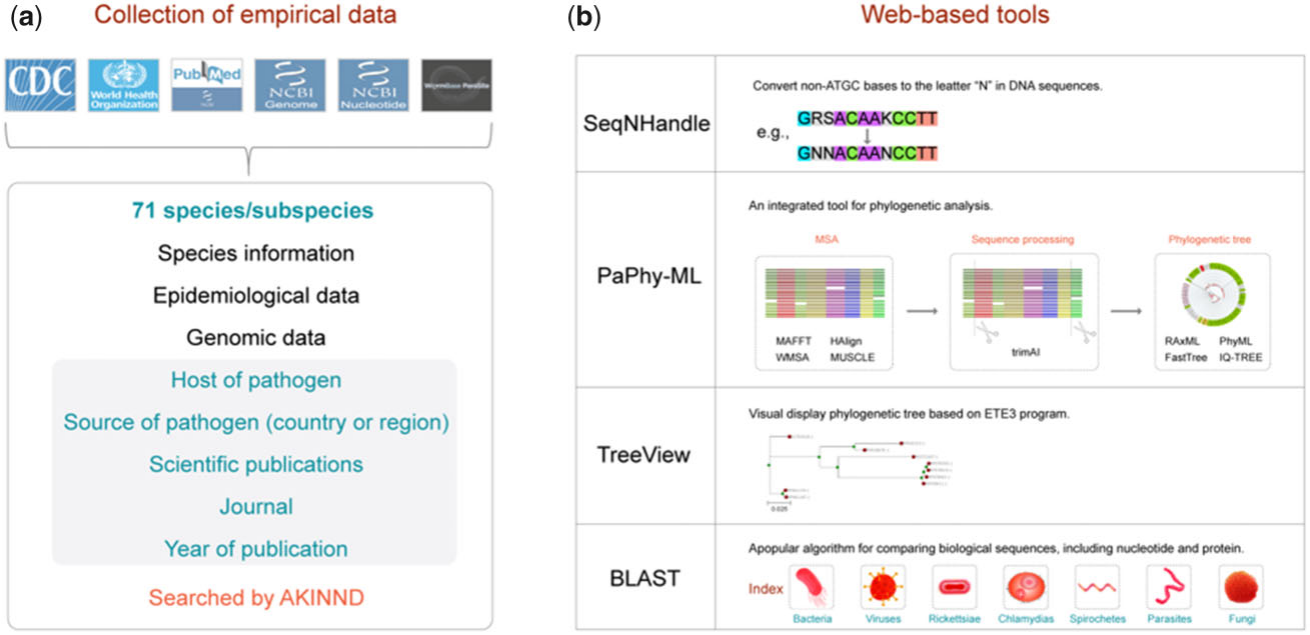
Graphical depiction of ZooPathWeb, which mainly contains the collection of empirical data (**a**) and the workflow of four web-based tools (**b**)

Although more than 200 types of zoonotic pathogens are currently recognized, research efforts should prioritize those that impose significant public health burdens on both human and animal populations. To obtain comprehensive datasets, we conducted information queries across multiple websites, including CDC, WHO, NCBI PubMed, NCBI Nucleotide and WormBase Parasite (Bolt *et al.*, 2018) (Fig. 1a). In this latest release, we have added zoonotic pathogens originating from 71 species/subspecies to ZooPathWeb (Supplementary Material). Additionally, we have gathered relevant pathogen information from diverse sources, encompassing basic details, epidemiological data, genomic data, host information, epidemic country/region, scientific publications, article journals and publication years, thereby providing broad coverage across different types of zoonotic pathogens.

Determining the reservoir hosts or vectors and understanding the population structure of zoonotic pathogens are of paramount importance for effectively preventing and controlling zoonoses. However, most zoonotic pathogens have a wide host range and are susceptible to strain variations that can be influenced by various factors, such as climate, geography and the environment. To obtain information on zoonotic pathogens and their molecular characterization, we conducted thorough literature searches. Within ZooPathWeb, we utilized the AKINND tool, developed specifically for this study, to extract relevant information from the NCBI Nucleotide database. We compiled a literature search library consisting of articles published within the last 30 years. Detailed descriptions of the webserver’s search processes and the specific functions of the AKINND tool are provided in Supplementary Figure S1. It should be noted that the search library may not include all articles pertaining to the zoonotic pathogens presented in this study, as the AKINND program may not be able to obtain certain qualifying information. A detailed explanation is provided in Supplementary Figure S2.

Our curated high-quality and comprehensive genomic datasets from established databases are now accessible for download in the ‘Download’ section. These datasets provide valuable resource for conducting phylogenetic analysis and identifying important population structures of zoonotic pathogens. Our web resource offers reliable information and serves as a specialized and unique resource in this field.

### 3.2 Web-based tools

In this study, we have developed four web-based tools, namely SeqNHandle, PaPhy-ML, TreeView and BLAST (Fig. 1b), which are valuable resources for researchers analyzing genomic data and identifying the molecular characteristics of zoonotic pathogens.

**SeqNHandle:** This web-based tool addresses issues arising from codon degeneracy or accidental errors in DNA sequencing, which can result in sequences containing multiple bases at the same position, also known as degenerate bases or ‘wobble’. The universal letters used to represent degenerate bases in a sequence are R, Y, M, K, S, W, B, D, H, V and N. SeqNHandle uniformly converts these degenerate bases to the letter N, improving the accuracy of multiple sequence alignment (MSA) and phylogenetic analysis of processed DNA sequences. Users can submit tasks by adding text or uploading a file in FASTA format. Upon completion, the full sequence text with degenerate bases converted to the letter N can be downloaded (see Supplementary Fig. S3).

**PaPhy-ML:** To identify the population structure of a pathogen, constructing a phylogenetic tree is necessary to determine the community relationships of species. In this study, we divided the PaPhy-ML program into two sections: MSA and Maximum-Likelihood (ML)-based phylogenetic analysis. Users have the option to select MSA software, including MAFFT v7.508 (Katoh and Standley, 2013), HAlign v3.0.0_rc1 (Tang *et al.*, 2022), WMSA v0.4.3 (Wei *et al.*, 2022) and MUSCLE v5.1 (Edgar, 2022). Typically, MSA results contain poorly aligned regions at both ends. To resolve this issue, we utilized the trimAI v1.4.1 (Capella-Gutierrez *et al.*, 2009) program to eliminate non-alignment segments. Additionally, users have the option to select ML-based phylogenetic analyzing software, including RAxML v8.2.12 (Stamatakis, 2014), PhyML v3.3.20190909 (Guindon *et al.*, 2010), FastTree v2.1.11 (Price *et al.*, 2010) and IQ-TREE v2.0.4 (Nguyen *et al.*, 2015). Zhou *et al.* (2018) systematically evaluated and compared these four programs using empirical genome-scale data matrices, revealing that IQ-TREE slightly outperforms the other three software in terms of accuracy. Regarding PaPhy-ML, users can freely choose MSA and phylogenetic analysis programs, and they can add text or files through the web version to analyze the molecular evolution characterization of pathogens. Users can access the results by downloading them as a text file or receiving them via email (see Supplementary Fig. S4).

**TreeView:** The results generated by PaPhy-ML rely on a phylogenetic tree program that generates a text file in the standard Newick tree format, commonly used for tree visualization. In this study, we have developed a web-based visualizer using the Python package ETE3 (Huerta-Cepas *et al.*, 2016). Users can conveniently visualize phylogenetic trees by either entering text or uploading a file (see Supplementary Fig. S5).

**BLAST:** BLAST is a fundamental tool for sequence alignment (Camacho *et al.*, 2009). It enables the comparison of the provided nucleotide or amino acid sequences with an indexed file using specified function options. In our study, we have developed a web server for BLAST, allowing users to input text or upload a file through the corresponding interfaces (Blastn Gene and Blastp Protein). Additionally, users can select a relevant species database and customize BLAST parameters for sequence alignment. The resulting output can be downloaded as a text file or delivered to a valid email (see Supplementary Fig. S6).

## Supplementary Material

vbad094_Supplementary_Data
