# Supplementary material for: ZooPathWeb: a comprehensive web resource for zoonotic pathogens
Source: Bioinform Adv. 2023 Jul 10;3(1):vbad094. doi: 10.1093/bioadv/vbad094 (PMC10351968; doi:10.1093/bioadv/vbad094)
Supplement: vbad094_Supplementary_Data [file vbad094_supplementary_data.docx]

**SUPPLEMENTARY INFORMATION**

**ZooPathWeb: A comprehensive web resource for zoonotic pathogens**

**Rui-Si Hu^1,2^, Xin Zhang^1,2^, Yanming Wei^1,3^**

^1^Yangtze Delta Region Institute (Quzhou), University of Electronic Science and Technology of China, Quzhou, Zhejiang 324003, China;

^2^Institute of Fundamental and Frontier Sciences, University of Electronic Science and Technology of China, Chengdu, Sichuan 610054, China;

^3^School of Computer Science and Technology, Xidian University, Xi’an, Shaanxi 710071, China.

*To whom correspondence should be addressed.

1. **Data collection…………………….…………………………………….…….…..**pp.2-3
2. **Backend Parameter Settings……………………..……..……….….…...…….....**pp.4-5
3. **Supplementary Figures………………………………………………………..…**pp.6-11
4. **Data collection**

According to the official report from the World Health Organization ([WHO](https://www.who.int/news-room/fact-sheets/detail/zoonoses)), there are more than 200 known types of zoonotic pathogens. Due to our close relationship with various animals in agriculture, approximately one-third of zoonotic pathogens pose a significant public health problem worldwide. Moreover, pathogens can evolve, leading to changes in their host adaptability. Some pathogens initially exist as zoonoses but later mutate into strains that primarily affect humans, such as the HIV virus and malaria parasites affecting humans.

In the present study, our primary objective was to collect data on zoonotic pathogens that are currently shared between humans and animals, with a significant impact on human health. As depicted in **Table 1**, we compiled a list of 71 species/subspecies of zoonotic pathogens whose hosts are shared by humans and animals, as of October 25, 2022. We selected high-quality genome data (the assembly should be complete at chromosome level and the genome sequence sufficiently long) for zoonotic pathogens, including viruses, bacteria, spirochetes, chlamydia, coxiella, fungi, and protozoan parasites, which we downloaded from the [NCBI Genome](https://www.ncbi.nlm.nih.gov/%20data-hub/genome/) database. Additionally, genomic data for helminth parasites were obtained from the [WormBase Parasite](https://parasite.wormbase.org/species.html) database.

For viral genomes, we first performed multiple sequence alignments using HAlign3 (Tang et al., 2022) and MAFFT (Katoh et al., 2013) software. Subsequently, degenerate bases were converted to the letter “N” using [SeqNHandle](http://112.124.26.17:39674/SeqNHandle/?name=SeqNHandle). Finally, the phylogenetic trees were constructed using the [PaPhy-ML](https://github.com/RuiSiHu/PaPhy-ML) following the usage of the evolutionary tree software RAxML (Stamatakis et al., 2014).

**Note that** in this web release, genome comparisons among spirochete bacteria, chlamydia, coxiella, fungi, and parasites were not conducted due to the limited sample size of genomic datasets and the current limitations of MSA software in processing large whole genomic data, especially for eukaryotic parasite and fungal pathogens. However, we anticipate that future advancements in comparative analysis quality, computational efficiency, and storage capacity of MSA software, along with the ability to perform genome alignments on a broader range of zoonotic pathogens, will enhance our understanding of population structure and evolutionary characteristics of zoonotic pathogens. Consequently, this will strengthen disease prevention and control efforts.

**Table 1.** A list of pathogens with hosts shared by humans and animals: collection and processing of empirical genomic data (October 25, 2022; [Download](http://112.124.26.17:39674/t/?name=Download&key=Category%E3%80%81Organism%E3%80%81Genome%E3%80%81Alignment%E3%80%81Tree%E3%80%81Information%E3%80%81Ref_genome%E3%80%81Ref_gene%E3%80%81Ref_protein&addkey=Category%E3%80%81Organism%E3%80%81Genome%E3%80%81Alignment%E3%80%81Tree%E3%80%81Information%E3%80%81Ref_genome%E3%80%81Ref_gene%E3%80%81Ref_protein&fujian=Genome%E3%80%81Alignment%E3%80%81Tree%E3%80%81Information%E3%80%81Ref_genome%E3%80%81Ref_gene%E3%80%81Ref_protein&fuwenben=&ismy=0)).

| **Category** | **Organism** | **No. of Genome** | **Tree** | **Information** | **Ref_genome** | **Ref_gene** | **Ref_protein** |
| --- | --- | --- | --- | --- | --- | --- | --- |
| Virus | *Chikungunya virus* | 846 | √ | √ | √ | √ | √ |
| Virus | *Dengue virus* | 4657 | √ | √ | √ | √ | √ |
| Virus | *Hantavirus* | 216 | √ | √ | √ | √ | √ |
| Virus | *Influenza A virus* | 3665 | √ | √ | √ | √ | √ |
| Virus | *Japanese encephalitis virus* | 432 | √ | √ | √ | √ | √ |
| Virus | *Marburg virus* | 89 | √ | √ | √ | √ | √ |
| Virus | *Monkeypox virus* | 387 | √ | √ | √ | √ | √ |
| Virus | *Nipah henipavirus* | 90 | √ | √ | √ | √ | √ |
| Virus | *Rabies lyssavirus* | 1786 | √ | √ | √ | √ | √ |
| Virus | *Rift valley fever virus* | 204 | √ | √ | √ | √ | √ |
| Virus | *Tick-borne encephalitis virus* | 164 | √ | √ | √ | √ | √ |
| Virus | *West Nile Virus* | 1716 | √ | √ | √ | √ | √ |
| Virus | *Zaire ebolavirus* | 574 | √ | √ | √ | √ | √ |
| Virus | *Zika virus* | 321 | √ | √ | √ | √ | √ |
| Bacterium | *Actinomyces* spp*.* | 30 |  | √ | √ | √ | √ |
| Bacterium | *Arcobacter* spp*.* | 7 |  | √ | √ | √ | √ |
| Bacterium | *Bacillus anthracis* | 100 |  | √ | √ | √ | √ |
| Bacterium | *Bordetella bronchiseptica* | 23 |  | √ | √ | √ | √ |
| Bacterium | *Brucella abortus* | 23 |  | √ | √ | √ | √ |
| Bacterium | *Brucella canis* | 8 |  | √ | √ | √ | √ |
| Bacterium | *Brucella melitensis* | 67 |  | √ | √ | √ | √ |
| Bacterium | *Brucella suis* | 30 |  | √ | √ | √ | √ |
| Bacterium | *Burkholderia mallei* | 27 |  | √ | √ | √ | √ |
| Bacterium | *Campylobacter coli* | 58 |  | √ | √ | √ | √ |
| Bacterium | *Campylobacter fetus* | 23 |  | √ | √ | √ | √ |
| Bacterium | *Campylobacter jejuni* | 261 |  | √ | √ | √ | √ |
| Bacterium | *Corynebacterium pseudotuberculosis* | 113 |  | √ | √ | √ | √ |
| Bacterium | *Corynebacterium ulceran* | 19 |  | √ | √ | √ | √ |
| Bacterium | *Erysipelothrix rhusiopathiae* | 11 |  | √ | √ | √ | √ |
| Bacterium | *Escherichia coli O157:H7* | 155 |  | √ | √ | √ | √ |
| Bacterium | *Francisella tularensis* | 60 |  | √ | √ | √ | √ |
| Bacterium | *Helicobacter* spp. | 350 |  | √ | √ | √ | √ |
| Bacterium | *Mycobacterium leprae* | 4 |  | √ | √ | √ | √ |
| Bacterium | *Mycobacterium tuberculosis* | 666 |  | √ | √ | √ | √ |
| Bacterium | *Pasteurella multocida* | 114 |  | √ | √ | √ | √ |
| Bacterium | *Salmonella bongori* | 12 |  | √ | √ | √ | √ |
| Bacterium | *Salmonella enterica* | 718 |  | √ | √ | √ | √ |
| Bacterium | *Vibrio parahaemolyticus* | 76 |  | √ | √ | √ | √ |
| Bacterium | *Yersinia pestis* | 58 |  | √ | √ | √ | √ |
| Spirochete | *Leptospira kobayashii* |  |  |  | √ | √ | √ |
| Chlamydia | *Chlamydia psittaci* |  |  |  | √ | √ | √ |
| Coxiella | *Coxiella burnetii* |  |  |  | √ | √ | √ |
| Fungus | *Coccidioides immitis* |  |  |  | √ | √ | √ |
| Fungus | *Coccidioides posadasii* |  |  |  | √ | √ | √ |
| Fungus | *Cryptococcus neoformans* |  |  |  | √ | √ | √ |
| Fungus | *Histoplasma capsulatum* |  |  |  | √ | √ | √ |
| Fungus | *Malassezia* spp*.* |  |  |  | √ | √ | √ |
| Fungus | *Microsporum* spp. |  |  |  | √ | √ | √ |
| Fungus | *Sporothrix schenckii* |  |  |  | √ | √ | √ |
| Fungus | *Trichophyton* spp. |  |  |  | √ | √ | √ |
| Parasite | *Ascaris suum* |  |  |  | √ | √ | √ |
| Parasite | *Blastocystis* spp. |  |  |  | √ | √ | √ |
| Parasite | *Clonorchis sinensis* |  |  |  | √ | √ | √ |
| Parasite | *Cryptosporidium parvum* |  |  |  | √ | √ | √ |
| Parasite | *Echinococcus granulosus* |  |  |  | √ | √ | √ |
| Parasite | *Echinococcus multilocularis* |  |  |  | √ | √ | √ |
| Parasite | *Fasciola gigantica* |  |  |  | √ | √ | √ |
| Parasite | *Fasciola hepatica* |  |  |  | √ | √ | √ |
| Parasite | *Giardia lamblia* |  |  |  | √ | √ | √ |
| Parasite | *Leishmania major* |  |  |  | √ | √ | √ |
| Parasite | *Neospora caninum* |  |  |  | √ | √ | √ |
| Parasite | *Plasmodium knowlesi* |  |  |  | √ | √ | √ |
| Parasite | *Schistosoma japonicum* |  |  |  | √ | √ | √ |
| Parasite | *Toxocara canis* |  |  |  | √ | √ | √ |
| Parasite | *Toxoplasma gondii* |  |  |  | √ | √ | √ |
| Parasite | *Trichinella spiralis* |  |  |  | √ | √ | √ |
| Parasite | *Trypanosoma brucei* |  |  |  | √ | √ | √ |
| Parasite | *Trypanosoma cruzi* |  |  |  | √ | √ | √ |

1. **Backend Parameter Settings**

The ZooPathWeb website was developed using the Django-based web application framework, which offers several advantages. These include its open-source nature, integration of commonly used tools and frameworks, and robust database functionality. **Figure 1** depicts our implementation strategy, which involves separating the frontend and backend in Django to present static webpages and retrieve backend data.

**
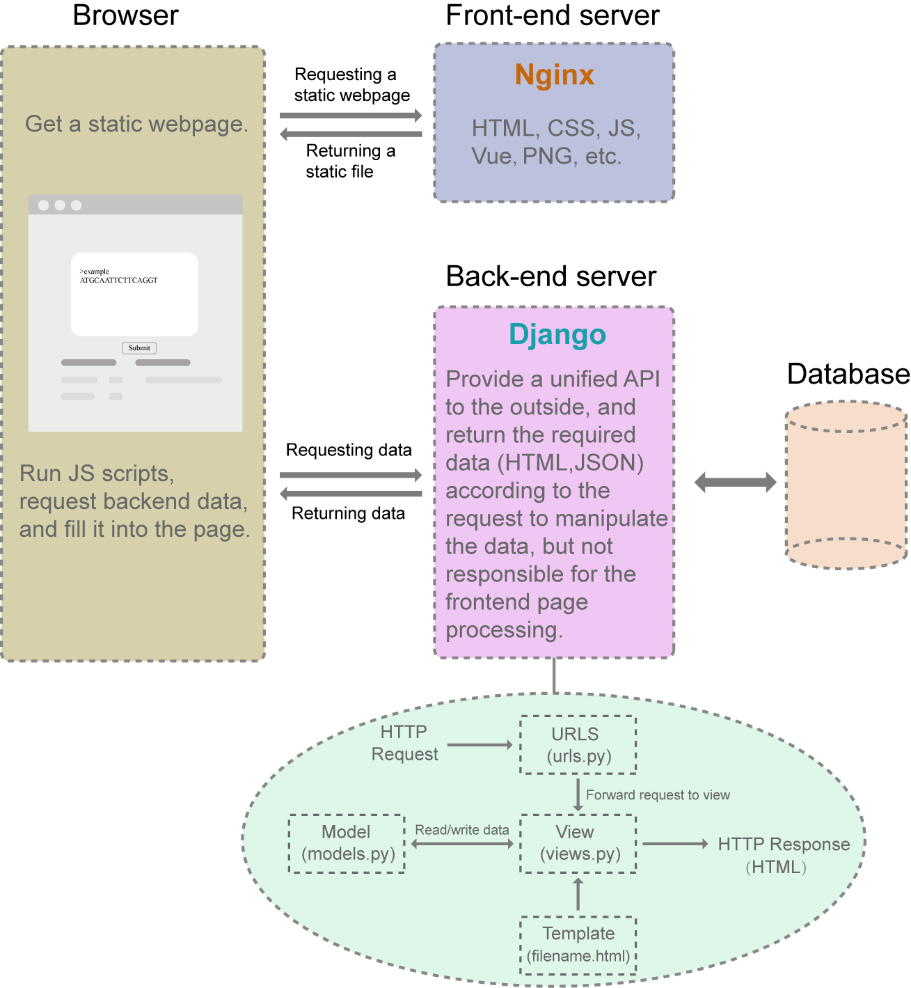
**

**Figure 1.** Diagram illustrating the frontend and backend separation of Django, as well as its basic framework structure.

For the current release, ZooPathWeb offers four online tools for identifying the population structure of pathogens: SeqNHandle, PaPhy-ML, TreeView, and BLAST.

The script for SeqNHandle is based on the idea proposed by [Tong Zhou.](https://github.com/malabz/MSATOOLS/tree/main/deal_N_tool) It is designed to convert all degenerate bases in a FASTA file to the letter "N" since sequence data containing "N" can be processed by most software. However, sequence data containing other degenerate bases may lead to software crashes. Supplementary Figure 3 provides detailed instructions on the usage of SeqNHandle, and the corrected script can be obtained from [GitHub](https://github.com/RuiSiHu/SeqNHandle/blob/main/SeqNHandle.py).

PaPhy-ML is an integrated tool for phylogenetic tree analysis. The website provides four MSA software for sequence alignment, including MAFFT v7.508 (Katoh and Standley, 2013) [*options: mafft in > out*], HAlign v3.0.0_rc1 (Tang, et al., 2022) [*options: halign -o in out*], WMSA v0.4.3 (Wei, et al., 2022) *[options: wmsa -i in -o out -T 2 -c 0.9], and MUSCLE v5.1 (Edgar, 2022) [options: muscle -align in -output out]*. The poor alignment parts for MSA are trimmed using trimAI v1.4.1 (Capella-Gutierrez, et al., 2009) *[options: trimal -nogaps -in in -out out -automated1]*. In addition, there are four software available for phylogenetic analysis based on the Maximum-Likelihood method, namely RAxML v8.2.12 (Stamatakis, 2014) *[options for nucleotide alignment: raxmlHPC -f a -x 123456 -p 123456 -s -m GTRGAMMA -N 1000 -T 20 -n txt -w; options for protein alignment: raxmlHPC -f a -x 123456 -p 123456 -s -m PROTGAMMALGX -N 1000 -T 20 -n txt -w]*, PhyML v3.3.20190909 (Guindon, et al., 2010) *[options for nucleotide alignment: phyml -i -d nt -b 1000 -m HKY85; options for protein alignment: phyml -i -d aa -b 1000 -m LG]*, FastTree v2.1.11 (Price, et al., 2010) *[options for nucleotide alignment: fasttree -nt -gtr in > out; options for protein alignment: fasttree -nt in > out]*, and IQ-TREE v2.0.4 (Nguyen, et al., 2015) *[options for nucleotide alignment: iqtree -s -mset HKY -m MFP -T AUTO; options for protein alignment: iqtree -s -mset LG -mfreq FU -m MFP -T AUTO]*. The detailed usage approach of PaPhy-ML can be found in **Supplementary Fig. 4**, and the raw script can be obtained on [GitHub](https://github.com/RuiSiHu/PaPhy-ML/blob/main/PaPhyML.py).

The TreeView is a tool used for visualizing and presenting phylogenetic trees. It is built upon the ETE3 program (Huerta-Cepas, et al., 2016). In the web server version, we provide four options for generating tree, which can be automatically generated in the backend based on parameters such as branch length, tree mode, branch vertical margin, and tree scale. **Supplementary Fig. 5** provides detailed instructions on the usage of TreeView, and the original script can be obtained from [GitHub](https://github.com/RuiSiHu/TreeView/blob/main/TreeView.py).

The use of the BLAST method for local alignment to identify species homology, including pathogens, is commonly employed. Our website offers online alignment services by inputting the sequence of a specific species. In the backend process, we have carefully selected high-quality reference genome sequences from both human and animal pathogens. We have established a BLAST genome index that enables users to achieve more accurate and effective alignment. For nucleotide alignment, the parameter options are as follows: *“[blastp -query input -out output -db nucleotide_index -outfmt -evalue -num_threads -max_target_seqs max_target_seqs]”*. Similarly, for protein alignment, the parameter options are “*[blastn -query input -out output -db nucleotide_index -outfmt -evalue -num_threads -max_target_seqs max_target_seqs]*”. Within the BLAST backend, we provide index files for seven distinct lineage species: Virus, Bacterium, Rickettsia, Chlamydia, Spirochete, Parasite, and Fungus. **Supplementary Fig. 6** provides detailed instructions on how to use BLAST, and the original script can be obtained from [GitHub](https://github.com/RuiSiHu/BLAST-Pathogens/blob/main/BLAST-Pathogens.py).

1. **Supplementary Figures**

**
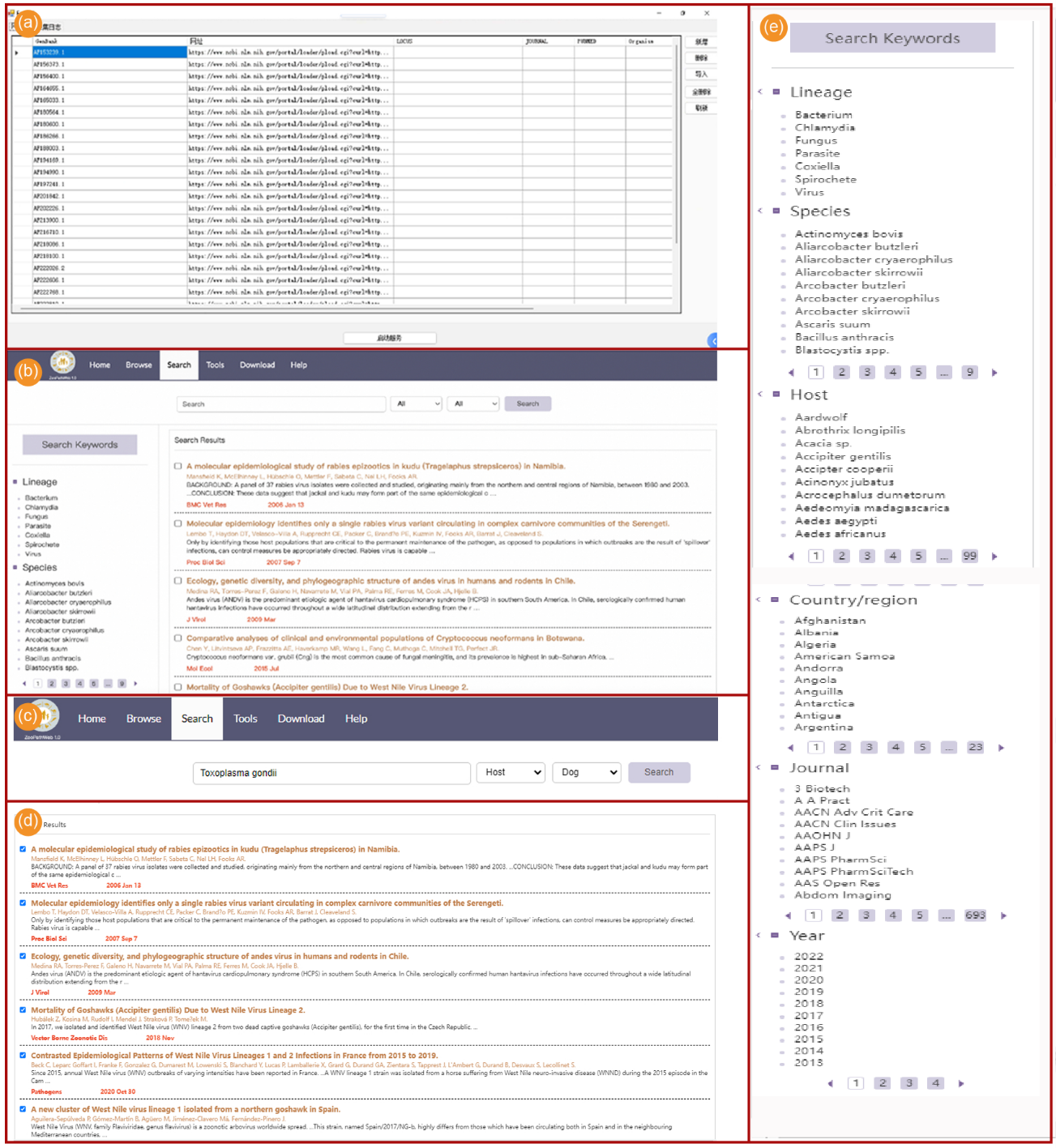
**

**Supplementary Fig. 1:** AKINND (Acquire Key Information from NCBI Nucleotide Database) workflow in the process of data collection and the development of a literature search library using Vue.js framework. In AKINND, users can input the GenBank ID of a pathogenic species and activate the service to automatically obtain basic information about the pathogen from the NCBI Nucleotide database (**a**). The literature on the species genome obtained will be packaged and built into a literature search library through the Vue.js framework in subsequent data processing (**b**). Users can specify keywords, such as lineage, species name, host, country or region, journal, and publication year, to conduct literature searches accordingly (**c**). In the search results, users can directly click on the literature link and jump to the NCBI PubMed page to view the detailed abstract or download the full text (**d**). Additionally, users can directly click on the corresponding keyword on the left side of the webpage for searching (**e**).

**
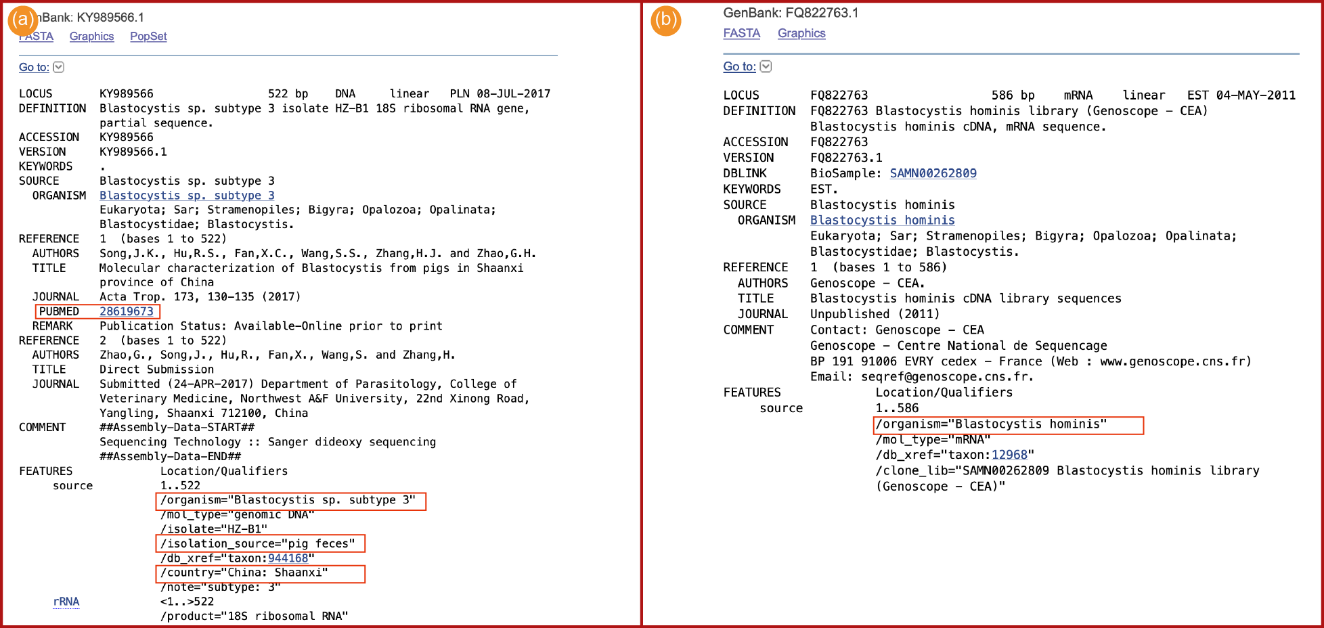
**

**Supplementary Fig. 2:** An example is provided to illustrate the situation encountered by AKINND during the acquisition of pathogenic data. After entering a specified GenBank ID in the NCBI Nucleotide database, the webpage will be redirected to the display page of the pathogenic gene sequence. AKINND's search strategy requires that the displayed information satisfies the criteria shown in (**a**), with important information including the PUBMED ID, species name, isolate source, and country or region (the red box in the figure). As shown in (**b**), if this information is not available, AKINND's search strategy will not be met. During the process of obtaining pathogenic species information, AKINND will automatically skip the GenBank ID that does not meet the search criteria and retrieve the next valid GenBank ID, which enable accelerate information acquisition and avoid interruptions.

**
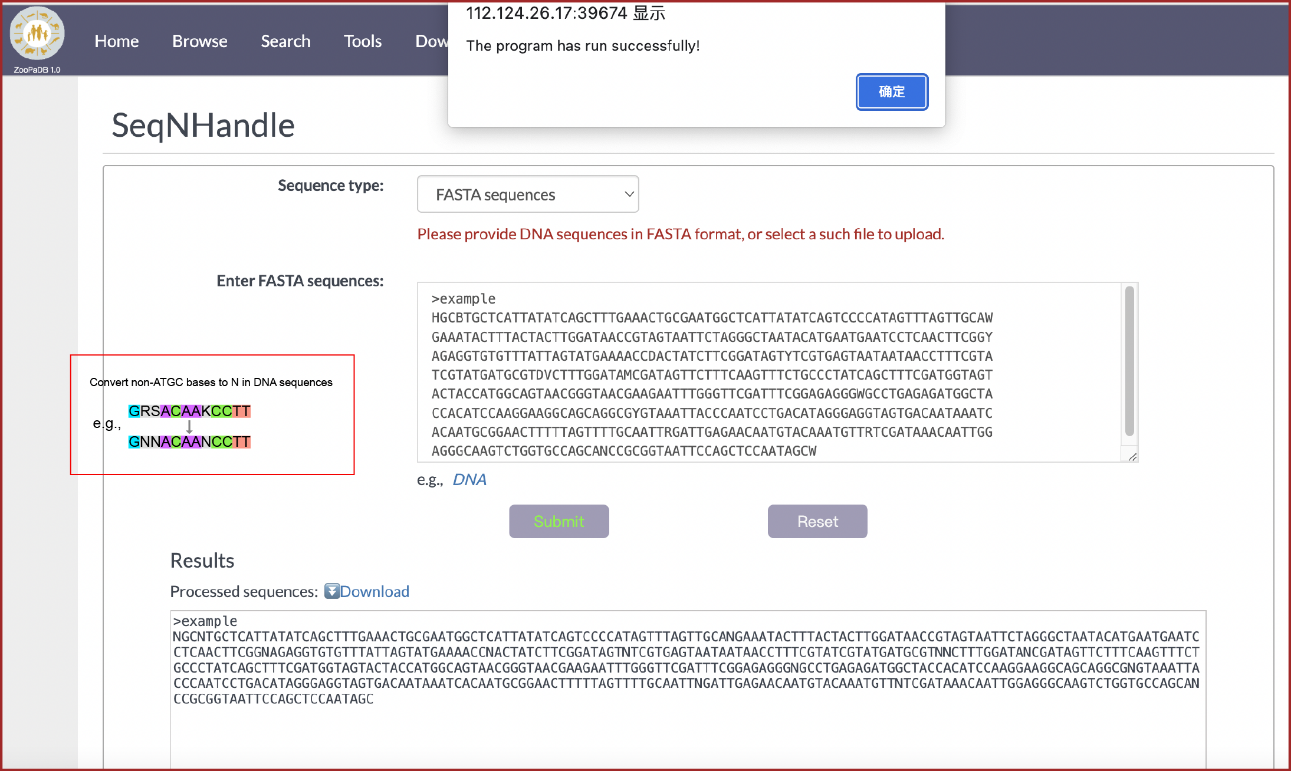
**

**Supplementary Fig. 3:** The usage method of SeqNHandle. Prior to performing multiple sequence alignment, preprocessing of degenerate bases in DNA sequences can effectively improve the efficiency of sequence alignment. Users can input FASTA sequences or upload a FASTA file and click "Submit" to obtain the processed sequences. Clicking "Download" will allow users to download the processed sequences in a text file, which can be used for multiple sequence alignment. To submit a new task, users can click the "Reset" button.

**
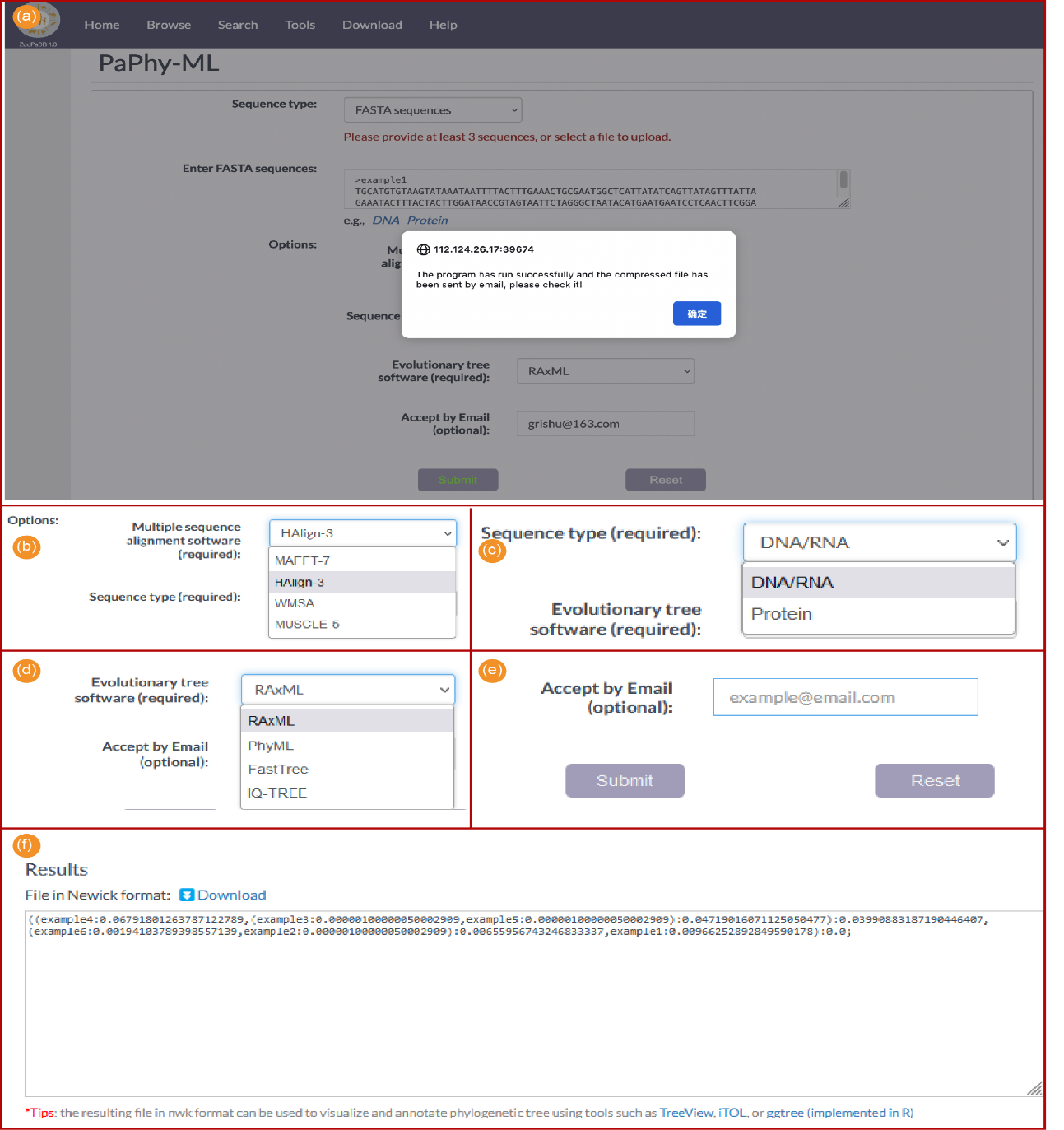
**

**Supplementary Fig. 4:** The usage method of PaPhy-ML, which is an effective tool for analyzing phylogenetic trees from multiple sequences. The sequences must be in FASTA format and can be directly pasted into the input box or uploaded as a FASTA file (**a**). We provide multiple sequence alignment options, including MAFFT-7, HAlign3, WMSA, and MUSCLE-5 (**b**). The input sequence type is also a required option, which can be either DNA/RNA sequences or protein sequences (**c**). Additionally, users must choose from RAxML, PhyML, FastTree, or IQ-TREE for the phylogenetic tree software (**d**). If the backend runtime is too long, users can choose to receive analysis results by entering a valid email address (**e**). Finally, the analysis results can be displayed in Newick format, which can be downloaded as a text file by clicking "Download". Users can click the "Reset" button if they want to submit a new task (**f**). The Newick format text can be visualized using phylogenetic tree visualization software, such as TreeView, iTOL, and ggtree.

**
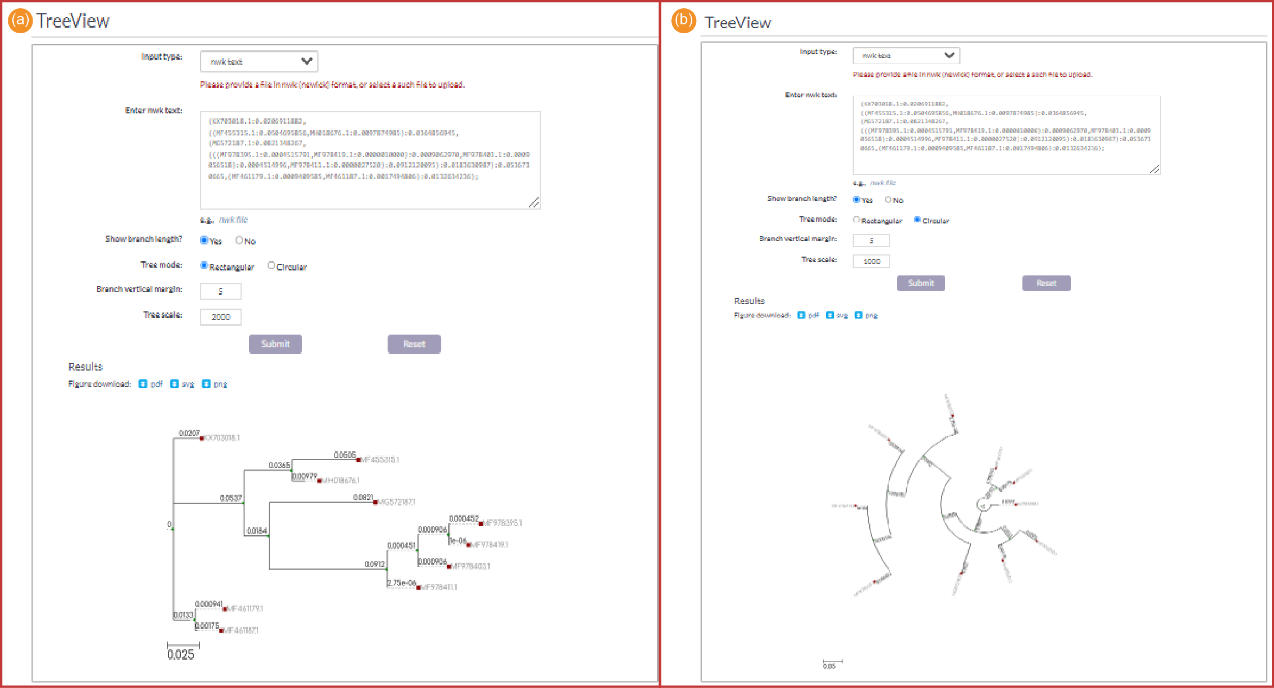
**

**Supplementary Fig. 5:** The usage method of TreeView. TreeView is capable of visualizing phylogenetic trees through systematic display. Users can input Newick formatted text or upload a text file, referencing example file information, namely nwk file. We provide four key options to adjust the image, namely Branch length, Tree mode, Branch vertical margin, and Tree scale. The default value for branch length is Yes, automatically adding branch length values at the upper end of the evolutionary tree branches. The default option for Tree mode is Rectangular (**a**), which can be changed to Circular for tree branch display if there are many branches (**b**). Branch vertical margin represents the length of the tree branches in the vertical direction, with a selectable value range of 1-100 and a default value of 5. Tree scale represents the length of the tree branches in the horizontal direction, with a selectable value range of 500-10000 and a default value of 2000. Clicking "pdf", “png”, or “svg” will allow users to download the phylogenetic tree figure.

**
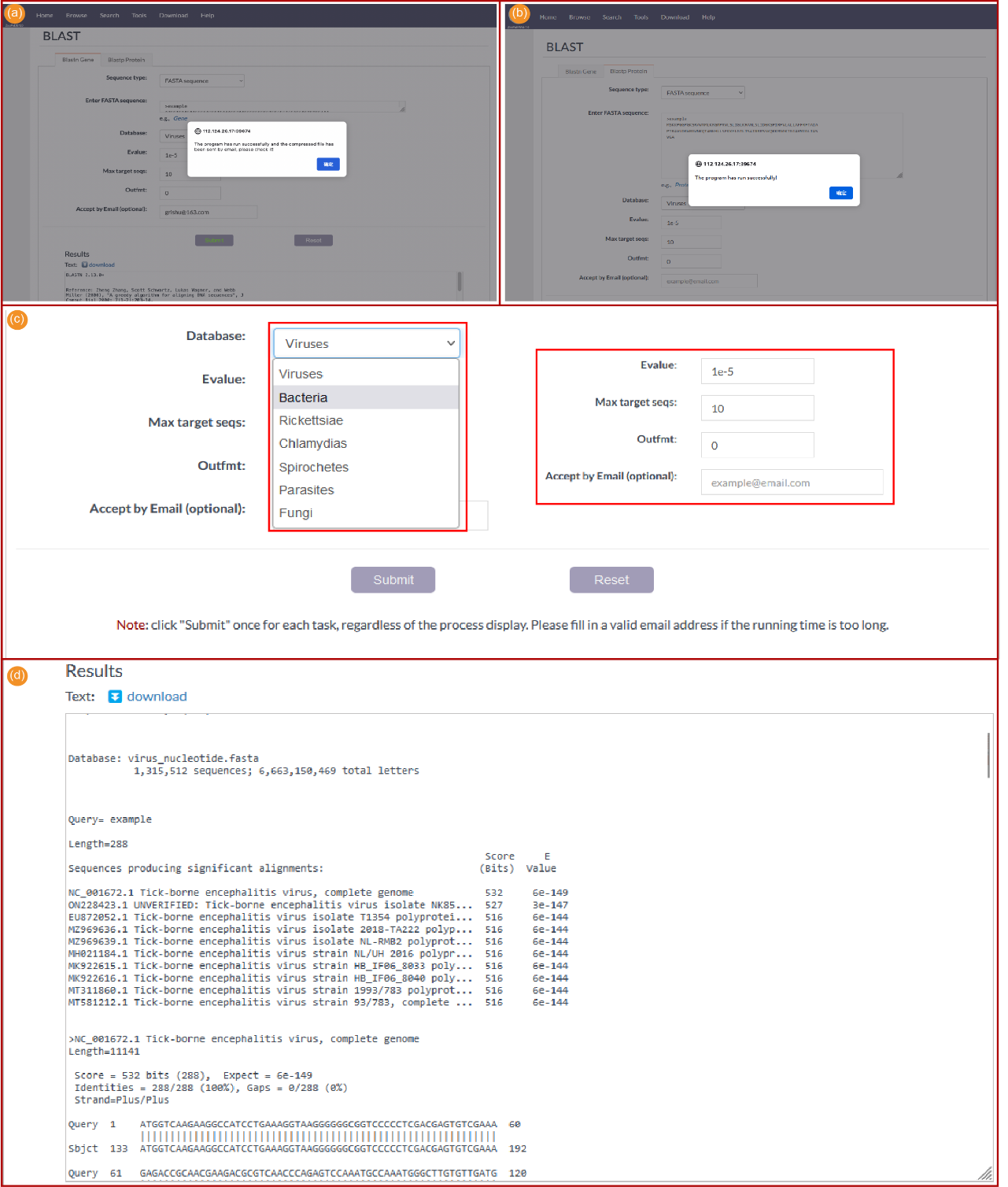
**

**Supplementary Fig. 6:** The usage method of BLAST-Pathogens. BLAST-Pathogens consists of two parts: one is the homology comparison of nucleic acid sequences (Blastn Gene) (**a**), and the other is the homology comparison of protein sequences (Blastp protein) (**b**). The operating methods for the two types of comparison are completely the same. Users can input FASTA sequences or upload a FASAT file. They need to select a corresponding pathogen database as the comparison index library, including Viruses, Bacteria, Rickettsiae, Chlamydias, Spirochetes, Parasites, and Fungi. In addition, they also need to select three important parameter values, including Evalue (default is 1e-5), Max target seqs (default is 10), and Outfmt (a selectable range of 0-17, default is 0, commonly used options are "-outfmt 0", "-outfmt 5", and "outfmt 6") (**c**). Click "Submit" to submit the task and click "Reset" to start a new task. Note that only one submission is required for each task, regardless of how the running status is displayed, the results will eventually be generated. Finally, the results can be displayed on the webpage, and clicking "Download" link can download the text (**d**).
